# Treatment of Dysentery

**Published:** 1851-06

**Authors:** 


*Treatment of Dysentery*, under some form, prevails in this country to a great extent during the summer and fall months, and for the last few years has been attended with a very great mortality. This may have been, to some extent, the result of erroneous views of the nature of the disease. That old and once popular pathology, which recognized "but one organ, the liver,— one disorder, the biliary,— and the remedy, calomel," we are gratified to think is rapidly giving way to more rational views. The *Hepatic* pathology at one time held almost undisputed sway: the liver was held responsible for the errors of almost every rebellious organ of the system, and for almost every deviation from the laws of health. It was a simple and convenient pathology, but therapeutical errors to which it gave rise were far from being of the same simple character. Its practical results were bad.— *Western Med. Chir. Journal*, (*Editorial.*)

